# A New Method for Chromosomes Preparation by ATP-Competitive Inhibitor SP600125 *via* Enhancement of Endomitosis in Fish

**DOI:** 10.3389/fbioe.2020.606496

**Published:** 2021-01-13

**Authors:** Wenting Xu, Yanxiu Mo, Yu He, Yunpeng Fan, Guomin He, Wen Fu, Shujuan Chen, Jinhui Liu, Wenbin Liu, Liangyue Peng, Yamei Xiao

**Affiliations:** ^1^State Key Laboratory of Developmental Biology of Freshwater Fish, Hunan Normal University, Changsha, China; ^2^College of Life Sciences, Hunan Normal University, Changsha, China; ^3^Department of Histology and Embryology, School of Basic Medical Science, Xiangnan University, Chenzhou, China

**Keywords:** SP600125, endomitosis, p53, spindle assembly checkpoint, chromosome

## Abstract

Previous studies have suggested that 1,9-Pyrazoloanthrone, known as SP600125, can induce cell polyploidization. However, what is the phase of cell cycle arrest caused by SP600125 and the underlying regulation is still an interesting issue to be further addressed. Research in this article shows that SP600125 can block cell cycle progression at the prometaphase of mitosis and cause endomitosis. It is suggested that enhancement of the p53 signaling pathway and weakening of the spindle assembly checkpoint are associated with the SP600125-induced cell cycle arrest. Using preliminary SP600125 treatment, the samples of the cultured fish cells and the fish tissues display a great number of chromosome splitting phases. Summarily, SP600125 can provide a new protocol of chromosomes preparation for karyotype analysis owing to its interference with prometaphase of mitosis.

**Graphical Abstract:**
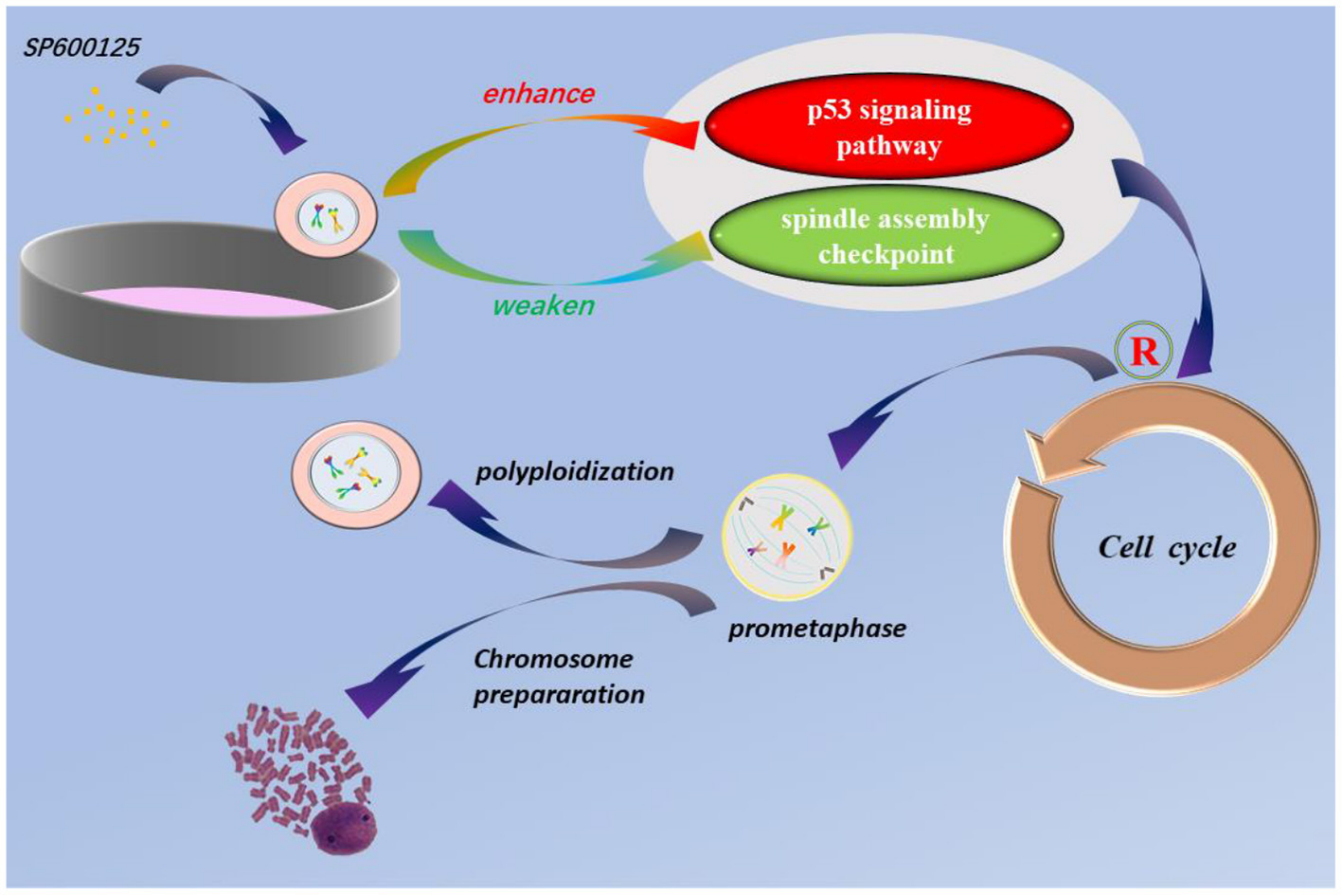
SP600125 blocks cell cycle at prometaphase and can be used for chromosomes preparation for karyotype analysis. The p53 signaling pathway and spindle assembly checkpoint are involved in SP600125-induced cell polyploidization.

## Introduction

1,9-Pyrazoloanthrone, known as SP600125, is an ATP-competitive inhibitor of kinases (Bennett et al., 2001; Chang and Karin, 2001). It is a highly selective inhibitor of c-Jun N-terminal kinase (JNK) and widely used to inhibit various JNK-mediated cell responses (Yarza et al., 2016; Dhanasekaran and Reddy, 2017; Gkouveris and Nikitakis, 2017). SP600125 can also affect cell apoptosis, progression of cancer, and pathological response in mammals (Nakaya et al., 2009; Kim et al., 2010). It was found that SP600125 plays important roles in maintaining stem cell characteristics of embryonic stem cells (Kook et al., 2013; Wei et al., 2014) and promoting efficiency of cell reprogramming into induced pluripotent stem cells (Yao et al., 2014; Chen et al., 2015; Ou et al., 2016).

Previous studies also showed that SP600125 inhibits cell proliferation in many human cancer cells and in embryonic stem cells by blocking cell cycle progression (Li et al., 2012; Mili et al., 2016; Hai et al., 2019). However, it is reported that effects of the SP600125 on cell cycle arrest are independent of its suppression of JNK activity, and that the knockdown of the *jnk* gene does not give rise to cell polyploidization (Kim et al., 2010; Zhou et al., 2016). Kim et al. (2010) suggested that SP600125 could suppress Cdk1 and induce endoreplication directly from G_2_ phase and indirectly inhibited the phosphorylation of Cdk1 and the persistence of Cdk2 activity. Some researchers also suggested that SP600125 treatment caused the induction of p21 and Cdk2 protein and resulted in G_2_/M arrest by inducing abnormal spindle microtubule dynamics (Moon et al., 2009, 2011). However, what is the phase of cell cycle arrest caused by SP600125 and the underlying regulation is still an interesting issue to be further addressed.

Our previous research showed that in the cell cycle of the caudal fin cells of diploid crucian carp (*Carassius auratus* L.) *in vitro*, treatment with 100 μM SP600125 causes a significant increase in tetraploid peak (4n) cells (Zhou et al., 2016; Mo et al., 2019). Indeed, a tetraploid cell line was generated from diploid fish cells by SP600125 cyclic treatment (Zhou et al., 2016). In this study, we intend to address how SP600125 induces polyploidization by arresting the cell cycle. It is indicated that SP600125-induced cell cycle arrest was found to appear from the prometaphase of mitosis. The result shows that SP600125 can provide a new way to prepare chromosome for karyotype analysis.

## Materials and Methods

### Cell Culture

The cells from the caudal fin of fish were cultured as done in our previous studies (Zhou et al., 2016; Mo et al., 2019). Specifically, the cells were cultured in Dulbecco's Modified Eagle's Medium (DMEM; Gibco, Life Technologies, CA, USA), supplemented with 100 U/ml penicillin, 100 μg/ml streptomycin (Invitrogen, Carlsbad, CA, USA), 10% fetal bovine serum (FBS; Invitrogen, Carlsbad, CA, USA), 0.1% 2-mercaptoethanol (2-ME; Invitrogen, Carlsbad, CA, USA), 1 mM sodium pyruvate (Invitrogen, Carlsbad, CA, USA), and 1 mM non-essential amino acids (Invitrogen, Carlsbad, CA, USA). Cells were grown in 5% (v/v) CO_2_ at 28°C, and all the used cells came from the 8th to the 10th passages.

SP600125 (C_14_H_8_N_2_O; Merck, Germany) was dissolved in dimethyl sulfoxide (DMSO 10%) as a 10 mM stock solution, which was then diluted to 100 μM for preparation. The operation of SP600125 cyclic treatment can be described as follows. Hundred micrometer SP600125 was first added to the culture medium where the cells reached 80–90% confluence. After SP600125 treatment for 48 h, the cells were then cultured in SP600125-free medium for 12 h. Finally, the cells were further treated again by SP600125 for 48 h.

### Cell Cycle Analysis by Flow Cytometry

To conduct cell cycle analysis, the cells were first digested into single-cell suspension by 0.25% trypsin. After being filtered through a 40 μm cell strainer, the cells were incubated with 2 μg/ml Hoechst 33342 (Invitrogen) and 50 μM Verapamil (Sigma) for about 15 min and then examined by flow cytometry (Sysmex Partec, Germany).

### Immunofluorescence

Immunofluorescence was conducted as described in our previous research (Peng et al., 2019). Specifically, the cells were fixed in 4% paraformaldehyde for 30 min and then treated with 0.3% Triton X-100 for 5 min. For non-specific blocking, the cells were incubated in 0.2% bovine serum albumin (Calbiochem, San Diego, CA, USA) for 15 min and then incubated with anti-α-tubulin antibody (dilution ratio was 1:100; GeneTex, Inc., North America, GTX628802) at 4°C for 12 h. The cells were stained with anti-mouse IgG fluorescent secondary antibody (dilution ratio was 1:200; Abways Biotechnology Co., Ltd., Shanghai, China, AB0132) for 2 h at room temperature. DNA was stained with Hoechst 33342 (Invitrogen). Fluorescence was imaged using OLYMPUS FV1200 confocal microscope (Olympus, Tokyo, Japan).

### Obtaining Transcriptome Data

We obtained mRNA sequencing (seq) data for caudal fin cells of crucian carp and SP600125-treated cells from the NCBI SRA database (SRR7640866, SRR7640867, SRR9964682, and SRR9964683) (Mo et al., 2019; Ren et al., 2020). Gene expression levels were calculated using the fragments per kilobases per million mapped reads (FPKM) method. The identification of differentially expressed genes (DEGs) between the control group and SP600125-treated cells was performed using the DESeq packages (DEGs of control and SP600125-treated cell samples were separately screened). Unigenes with false discovery rate (FDR) ≤ 0.001 and |log2Ratio| ≥2 were considered as DEGs for further analysis. Gene Ontology (GO) enrichment analysis was carried out with the Blast2 GO v2.5.0 software. All DEGs were mapped to terms in the Kyoto Encyclopedia of Genes and Genomes (KEGG) databases, and significantly enriched KEGG terms were searched for. GO terms with FDR <0.05 were considered to be significantly enriched.

### Quantitative Real-Time Polymerase Chain Reaction

The quantitative real-time polymerase chain reaction (qRT-PCR) was conducted as described in our previous research (Mo et al., 2019). All the qRT-PCR primers used in this study are listed in Supplementary Table 1. The used amplification conditions are stated as follows: 50°C for 2 min and 95°C for 10 min, followed by 40 cycles at 95°C for 15 s and 60°C for 1 min. The average threshold cycle (Ct) was calculated for each sample using the 2^−ΔΔCt^ method and normalized to β*-actin*. Finally, melting curve analysis was done to validate the specific generation of the expected product. For each sample, the qRT-PCR analysis was conducted three times. The reaction was carried out using the Prism 7500 Sequence Detection System (Applied Biosystems, Foster City, CA, USA) with a miScript SYBR Green PCR kit (Qiagen, Valencia, CA, USA).

### Western Blot Analysis

The cells were lysed with cold RIPA Lysis buffer (Gibco, USA) and centrifuged for 10 min at 10,000 rpm. The protein concentration was measured with a BCA Protein Assay kit (Thermo Scientific Pierce, Rockford, IL, USA). Total proteins were separated by 12% SDS polyacrylamide gel electrophoresis and transferred to a polyvinylidene difluoride membrane (Millipore, Bedford, MA, USA). The membrane was blocked with 5% (w/v) non-fat milk in TBS containing 0.05% Tween-20 (TBST) for 1 h at 37°C and then incubated with primary antibodies, including anti-p53 (Abcam, USA), anti-MDM2 (Bioworld, USA), anti-p21 (Bioworld, USA), anti-Mad2L1 (Bioworld, USA), anti-CDC20 (Bioworld, USA), anti-BUB1 (Proteintech, USA), anti-Flag-tag (Proteintech, USA), and anti-β-ACTIN (Abcam, USA), overnight. Membranes were then washed with TBST three times and were finally incubated with the horseradish peroxidase labeled anti-mouse (or anti-rabbit) IgG secondary antibody (dilution ratio was 1:5,000) for 40 min. Densitometric analysis was performed using Image Pro-Plus 6.0 software (Media Cybernetics, Silver Spring, MD, USA).

### PFT-α Incubation of Cells and Cell Viability Determination

Pifithrin-α (C_16_H_18_N_2_OS.HBr, PFT-α) was used as an inhibitor of p53 (Komarov et al., 1999). Cell viability was determined using the cell counting kit-8 (CCK-8) reagent according to the manufacturer's instructions (Takara Bio Inc., Shiga, Japan). The cells were seeded at 5 × 10^3^ cells per well in 96-well plates and then were incubated for the next 2 h after addition of 10 μl CCK-8.

### Construction of a Recombinant Adenovirus Vector for MAD2 Overexpression

The mad2 gene open reading frame was obtained from a *C. auratus* fin cell line by PCR amplification using the forward primer 5′-ATGTCGAAGACGCTGAAG-3′ and the reverse primer 5′-TCACATGGAGTTTGTCCTC-3′. The mad2 gene fragment was sub-cloned into adenovirus vector pMT85 (pAd-mCMV-GFP-3Flag-pA) at XbaI and ClaI sites. After identification using restriction enzymes, plasmid pMT85 was linearized and subsequently transformed into E. coli DH5α. The transformants were sequenced after identification by colony PCR. Finally, target plasmids using the AdMax adenoviral vector system (Stratagene, La Jolla, CA, USA) and helper plasmids (pBHGloxΔE1, 3Cre; Microbix, Canada) were constructed according to the manufacturer's instructions. Target and helper plasmids were co-transfected into HEK293 cells (American Type Culture Collection, Manassas, VA, USA) for virus packaging. Virus particles were subsequently purified and titrated by Western blot.

Virus transfection was conducted in the cells with 60% confluence in 24-well plates. The MAD2-OE group was transfected pAd-mCMV-MAD2-GFP-3-Flag-pA [multiplicity of infection (MOI) was 1,200] and cultured in 5% (v/v) CO_2_ at 28°C. The negative control group was transfected pAd-mCMV-GFP-3-Flag-pA, whereas for the blank control group, there was no any transfection.

### Karyotyping

The fin cells of fish were cultured in a complete growth medium with 5.0% (v/v) CO_2_ at 28°C. The cells were pre-treated with 100 μmol/L SP600125 for 22–28 h, digested into single-cell suspension by 0.25% trypsin, and then swollen with a hypotonic solution of 0.0375 mol/L KCl for 40–60 min. Cells were fixed twice with cold Carnoy's fixative (methanol: glacial acetic acid = 3:1, v/v) for 40 min each time, concentrated by centrifugation, and then dropped onto the cold slides. The chromosomes were stained with 5% Giemsa solution (Solarbio Inc., USA) for 30 min.

Fish were injected with 5–12 μg/g (body weight) phytohemagglutinin (PHA; Yuanye Biotechnology Co., Ltd., Shanghai, China) three times at intervals of 12–15 h. Then, they were injected with SP600125 (0.5–1.5 μg/g body weight). After 1–2 h, fish were anesthetized with 100 mg/L MS-222 (Sigma-Aldrich, St. Louis, MO, USA) before dissection, and their kidneys were snipped in saline. The kidney cells were treated with a hypotonic solution of 0.075 mol/L KCl for 40–60 min. The remaining steps in preparing chromosome spreads were the same as for the cultured cells (see above). About 50 metaphase phases of each sample were counted under optical microscopes.

### Statistical Analysis

All data were expressed by mean ± standard deviation. Statistical analysis was performed using the Student's *t*-test for comparison of two groups or one-way analysis of variance (ANOVA) for comparison of more than two groups, followed by the Tukey's multiple comparison tests. For multiple testing, the Bonferroni *post hoc* test of P values was performed. Statistical calculation was performed using GraphPad Prism data representing the means of three independent experimental results with a given alpha value of 0.05 and a given confidence level of 95%. The level of statistical significance was highlighted with asterisks in the figures as follows: *P* > 0.05, not significant; P < 0.05 (^*^) was considered statistically significant; P < 0.01 (^**^) was considered very significant; and P < 0.001 (^***^), extremely significant.

## Results

### SP600125 Blocks Cell Cycle Progression at Prometaphase of Mitosis

**Figure 1:**
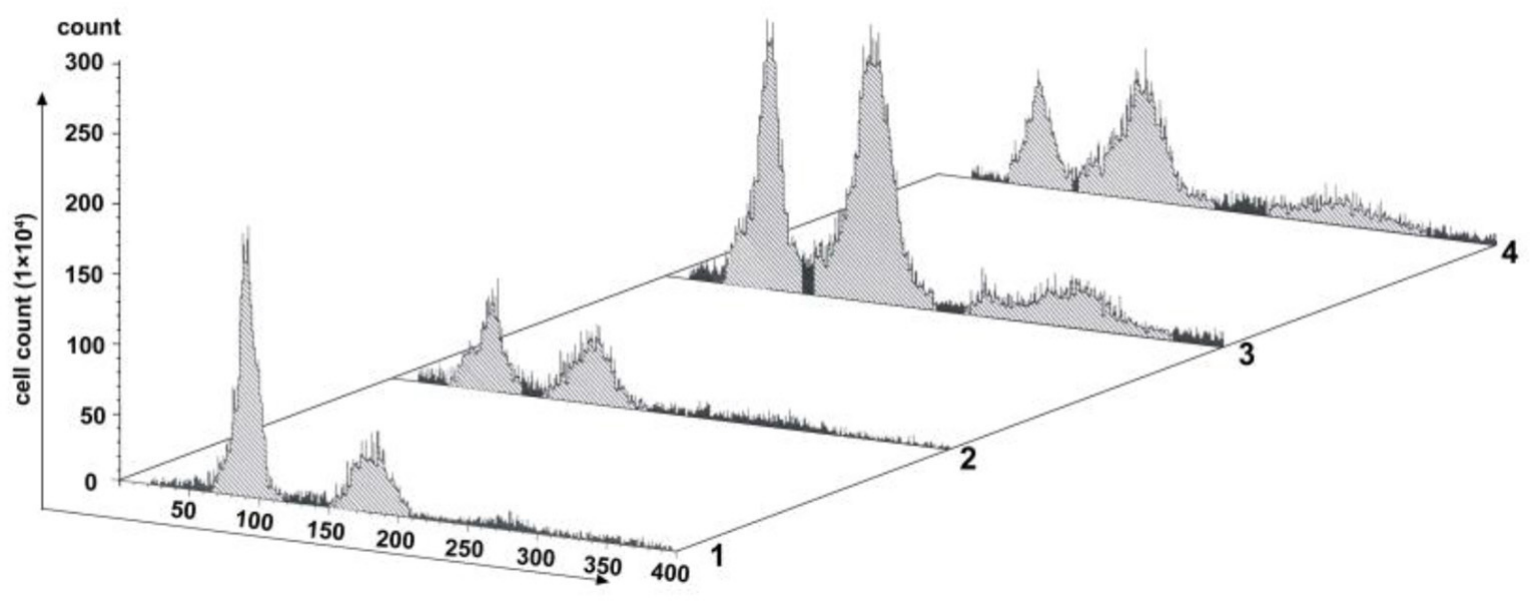
Flow cytometry analysis of the proliferation variation of SP600125-treated cells. Lane (1) showed the control group (cultured without SP600125); Lanes (2)–(4) showed the SP600125-treated groups: the cells treated by SP600125 for 48 h (Lane 2), SP600125-free medium cultured for the next 12 h (Lane 3), and then SP600125 treated again for a further 48 h (Lane 4).

Changes of cell proliferation were investigated at the process of SP600125 cyclic treatment by flow cytometry. As shown in Figure 1, after 48 h of SP600125 treatment, the proportion of diploid (2n) peak cells decreased from 41.54 to 23.44%, whereas that of tetraploid (4n) peak cells increased from 21.23 to 30.14% (Lanes 1 and 2 in Figure 1). When the cells were cultured for another 12 h in a SP600125-free medium, the proportions of the 2n and 4n peak cells were 22.87 and 38.94%, respectively. Moreover, there was 15.26% of octoploid (8n) peak cells (Lane 3 in Figure 1). When the cells were further treated by SP600125 for the next 48 h, the proportions of the 2n, 4n, and 8n peak cells were 18.04, 38.92, and 14.94%, respectively (Lanes 3 and 4 in Figure 1). The results confirmed that SP600125 could significantly block cell cycle and induce polyploidy for the fish cells.

**Figure 2:**
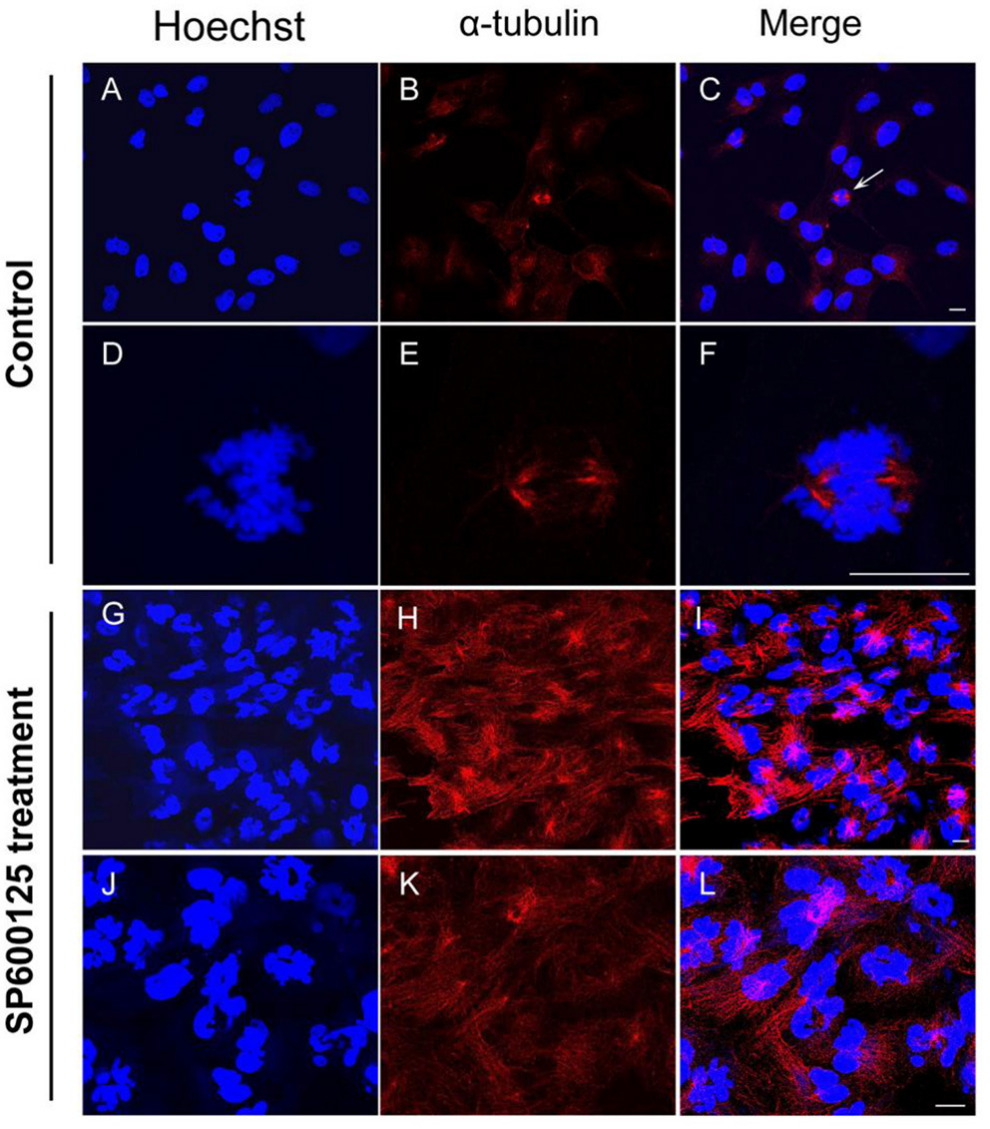
Cytological observation of SP600125-treated cells. **(A–F)** The control group (cultured without SP600125) and **(G–L)** the SP600125-treated group (SP600125 treatment for 48 h). Red regions showed the existence of tubulin using immunofluorescence staining. DNA (blue) was stained with Hoechst 33342. The white arrow in **(C)** showed the metaphase cells in the control group, and its enlarged parts were shown in **(D–F)**. Scale bars represent 10 μm.

By immunofluorescence staining, it was observed that the majority of the cells were at the prometaphase of mitosis after SP600125 treatment (Figures 2G–I). The nuclear membrane of these cells disappeared, the chromosomes were scattered, and spindle microtubules were visible (Figures 2J–L). However, in the control group (cultured without SP600125), the mitotic phase could be observed in few cells, and about 90% of the cells with intact nucleus appeared (Figures 2A–F).

### Expression Levels of p53 Relevant Cell Cycle Checkpoint Genes in SP600125-Treated Cells

**Figure 3:**
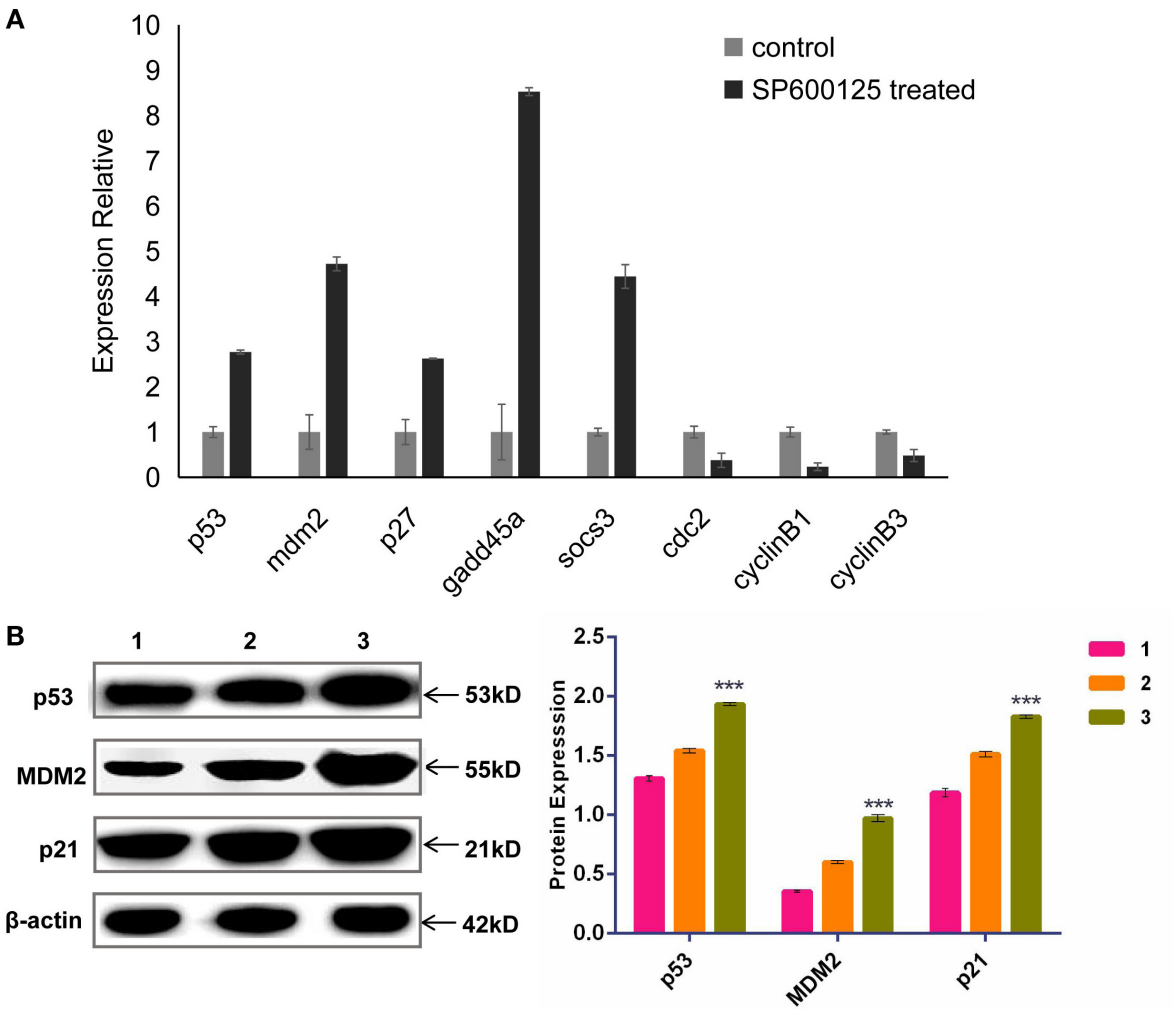
Expression levels of p53 pathway genes in SP600125-treated cells. **(A)** The mRNA levels of p53-relevant genes detected by qRT-PCR. **(B)** The expressed levels of p53 pathway detected by Western blot, where “1” represents the control group (being cultured without SP600125), “2” represents the cells treated by SP600125 for 48 h, and “3” stands for the cells being treated first by SP600125 for 48 h and then being further cultured for 12 h in SP600125-free medium. Each bar represents the mean ± SD of three independent experiments (*P < 0.05).

To examine any change in the global transcriptomic profile after SP600125 treatment, transcriptomes were obtained from the NCBI SRA database (Mo et al., 2019; Ren et al., 2020). Annotated by KEGG, a number of the significant DEGs identified between the SP600125-treated cells and control fish fin cells were associated with the cell cycle (3.52%), and this group was especially enriched in cell cycle checkpoint genes (Supplementary Table 2). For example, 1.01% of DEGs were related to the p53 signaling pathway. The qRT-PCR results demonstrated that the mRNA levels of p53 pathway genes, such as *mdm2, p53, p27, gadd45*α, and *socs3*, significantly increased after SP600125 treatment, whereas those of the cyclin–Cdk complex genes (*cyclin B1/3* and *cdc2*) were down-regulated (Figure 3A). The Western blot analysis also confirmed that there was significant up-regulation of p53, MDM2, and p21 in the SP600125-treated cells (Figure 3B).

**Figure 4:**
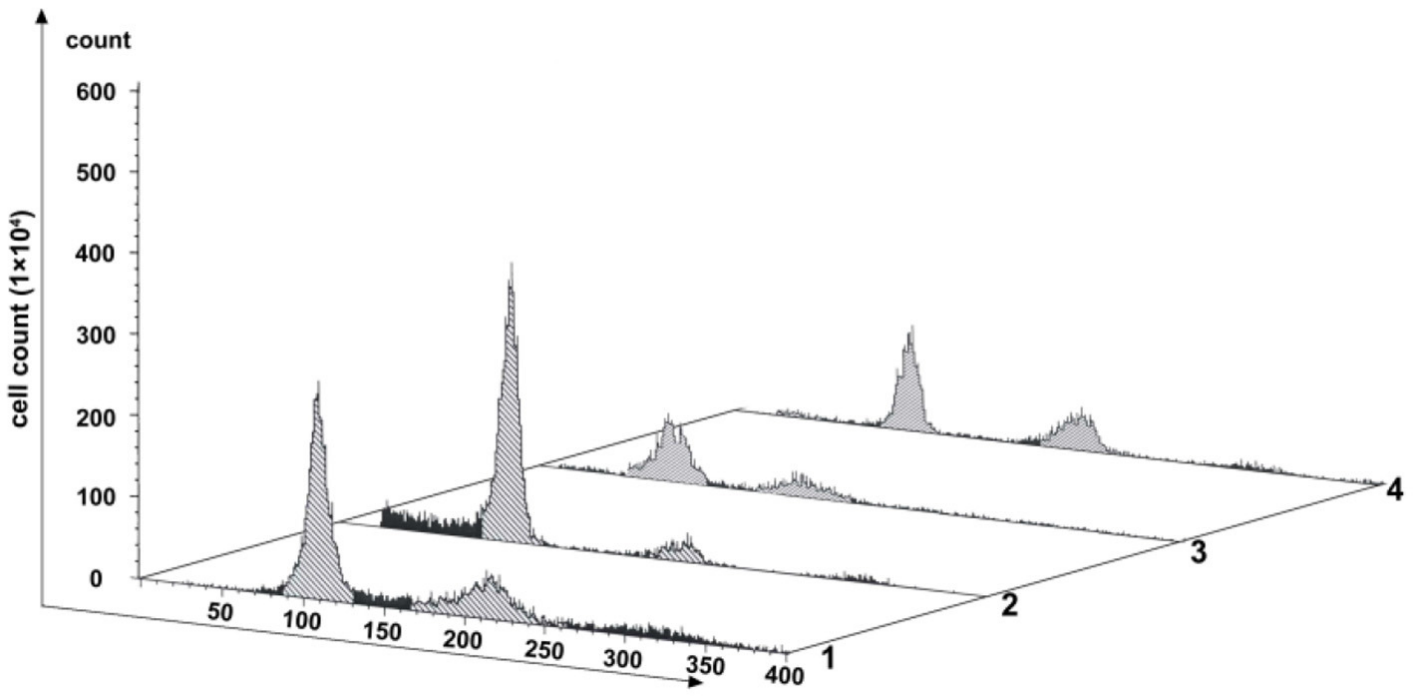
Effects of PFT-α on SP600125-treated cells. Flow cytometry analysis of the proliferation variation of cells. Lane 1 represents the control group (cultured without SP600125). Lane 2 stands for the PFT-α group (cells pretreated by PFT-α for 24 h. Lane 3 shows the cells first being cultured with PFT-α-free medium for 12 h and then being further treated by SP600125 for 48 h. Lane 4 shows the cells which the obtained cells in Lane 3 were further cultured in SP600125-free medium for another 12 h.

PFT-α is a transcriptional inhibitor of p53 and is known to protect against a variety of p53-mediated genotoxic agents (Komarov et al., 1999). Combined with cell viability determination and cell cycle blocking test, 40 μM PFT-α was used to treat the fish cells (named the PFT-α group) (Supplementary Figure 1 and Figure 4). PFT-α was added to the culture medium where the cells reached 80–90% confluence. The experimental results were stated as follows. (1) After PFT-α treated for 24 h, the cells were predominantly in G_1_ phase (about 64.14% cells), and there were 8.04% of the 4n peak cells (Lane 2 in Figure 4). In contrast, there were 41.54% of G_1_ cells and 21.23% of the 4n peak cells in the control group (Lane 1 in Figure 4). (2) Pretreated first by PFT-α for 24 h and PFT-α-free culture for another 12 h, then treated by SP600125 for the next 48 h, 46.69% of the cells were in G_1_ phase, and there were 21.52% of the 4n peak cells (Lane 3 in Figure 4). After further culturing these PFT-α group cells for 12 h in SP600125-free medium, 39.36% of these cells were in G_1_ phase, and there were 26.86% of the 4n peak cells (Lane 4 in Figure 4). Clearly, this proportion of the 4n peak cells is still lower than that of the SP600125-treated group (38.94%) (Lane 3 in Figure 1). These results indicated that weakening the activation of p53 can reduce cell cycle arrest in the SP600125-treated fish cells.

### Spindle Assembly Checkpoint Being Involved in SP600125-Induced Cell Cycle Arrest

**Figure 5:**
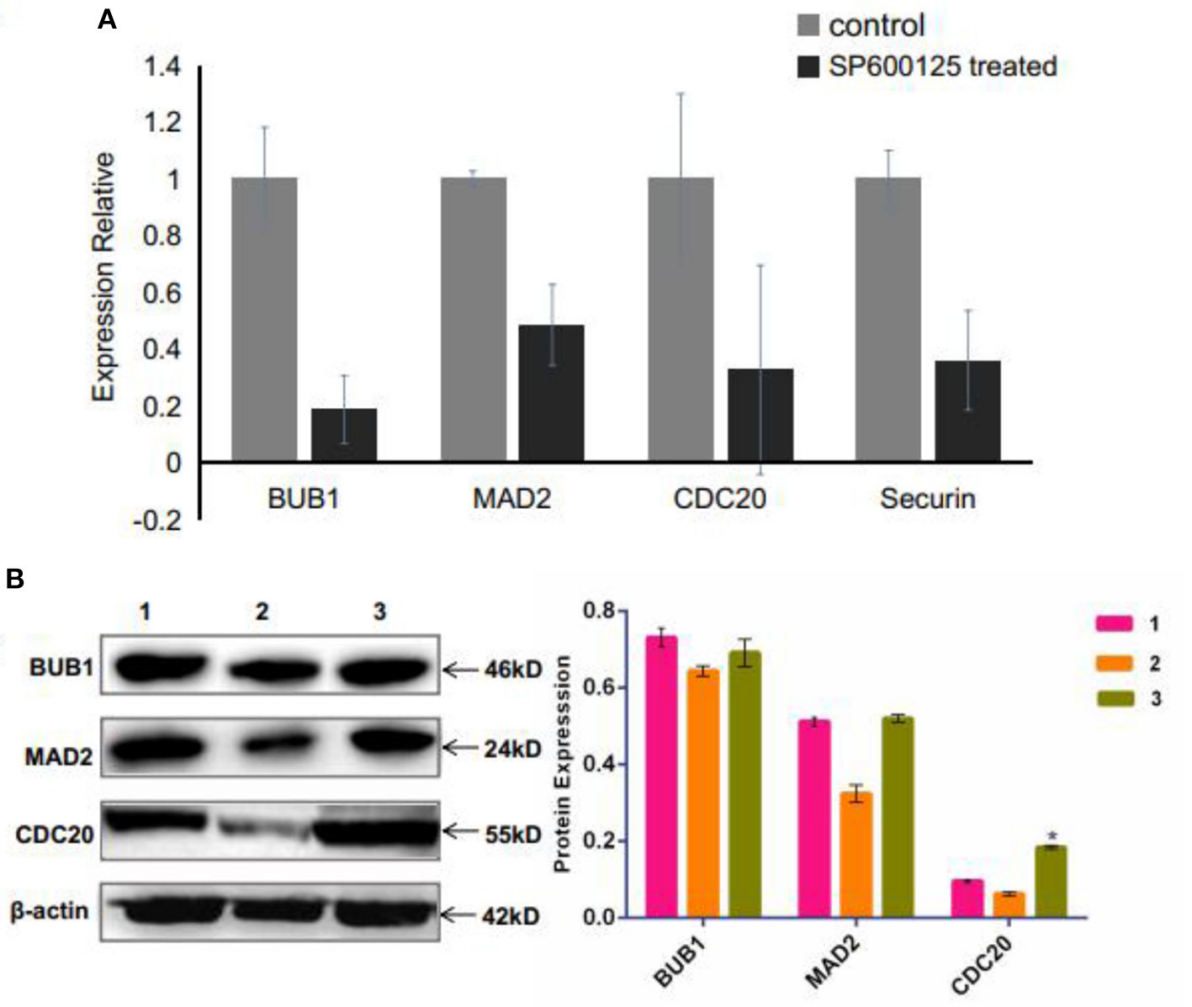
Effects of SP600125 on the expression levels of spindle assembly checkpoint genes. **(A)** qRT-PCR analysis for the differentially expressed *BUB1, MAD2*, and *CDC20* genes between the control group and the SP600125-treated cells. **(B)** Western blot analysis of expression levels of BUB1, MAD2, and CDC20 upon SP600125 treatment, where “1” represents the cells in the control group (without SP600125), “2” stands for the cells treated by SP600125 for 48 h, and “3” represents the cells first being treated by SP600125 for 48 h, then by SP600125-free culture for the next 12 h, and further by SP600125 treatment for another 48 h. Each bar represents the mean ± SD of three independent experiments (*P < 0.05).

The spindle assembly checkpoint (SAC), including mitotic arrest deficient (MAD), budding uninhibited by benzimidazole (BUB), and CDC20, is an active signal produced by improperly attached kinetochores (Nicklas, 1997). The RNA-seq and qRT-PCR results showed that the mRNA levels of *MAD2, BUB1*, and *CDC20* were remarkably down-regulated in the SP600125-treated cells (Supplementary Table 2 and Figure 5A). Western blot analysis also demonstrated that the expression levels of MAD2 and CDC20 reduced in the SP600125-treated cells (Figure 5B).

**Figure 6:**
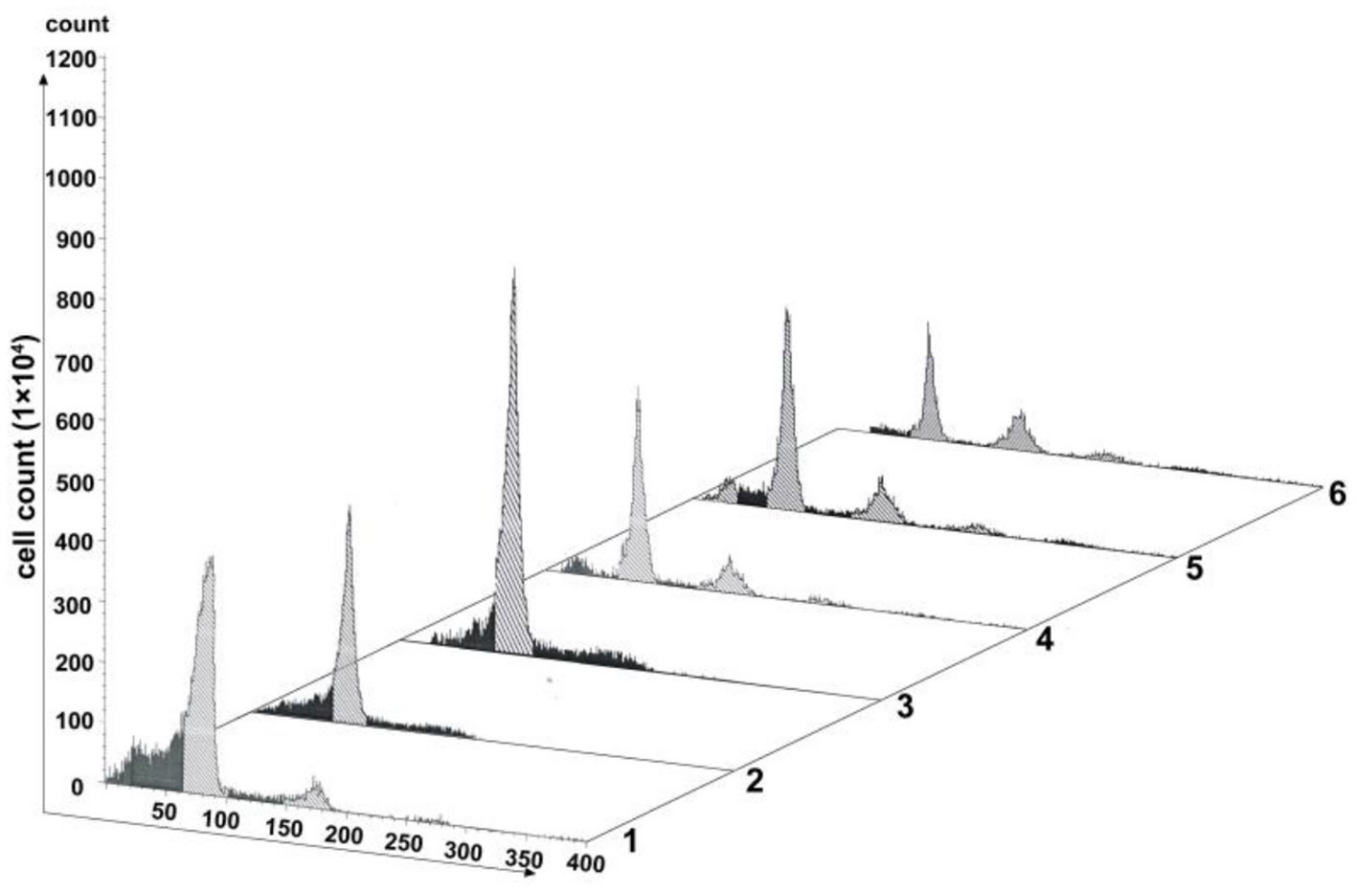
Analysis of cell proliferation by flow cytometry after transfection with pAd-MAD2. Lane 1 represents the normal cultured group. Lane 2 represents the negative control group. Lanes from 3 to 6 represent the overexpressed MAD2 groups (OE-MAD2): the cells being transfected with pAd-mCMV-MAD2-GFP-3-Flag-pA for 72 h (Lane 3), the cells being treated with SP600125 for the next 48 h (Lane 4), the cells continued to be further cultured in SP600125-free medium for 12 h (Lane 5), and the cells being further cultured using SP600125 treatment for another 48 h (Lane 6).

As one key factor of the SAC, MAD2 protein localizes to nuclei in a cell cycle-dependent manner (Kitagawa and Rose, 1999). We generated a pAd-mCMV-MAD2-GFP-3-Flag-pA vector for overexpressing *mad2* (Supplementary Figure 2). Virus transfection was conducted in cells with 60% confluence. As shown in Figure 6, transfection with pAd-mCMV-MAD2-GFP-3-Flag-pA for 72 h, about 59.64% of the OE-MAD2 cells were in G_1_ phase (Lane 3 in Figure 6), and there were 58.55% of the G_1_ cells in the blank control group and 63.83% of the G_1_ cells in the negative group (Lanes 1 and 2 in Figure 6). No any 4n peak cells were observed both in the OE-MAD2 group and in the negative group (Lanes 2 and 3 in Figure 6). In contrast, there were 7.02% of the 4n peak cells in the control group (Lane 1 in Figure 6). After SP600125 treatment for another 48 h, the proportion of the 4n peak cells in the OE-MAD2 group increased to 2.74%, and there were 53.52% of the cells in G_1_ phase (Lane 4 in Figure 6). When these cells were further cultured in SP600125-free medium for the next 12 h, the proportion of the 4n peak cells in the OE-MAD2 group increased to 4.43%, and that of the cells in G_1_ phase was 53.64% (Lane 5 in Figure 6). Even after an additional 48 h of SP600125 treatment again, the proportion of the 4n peak cells in the OE-MAD2 group was 8.54%, whereas that of the cells in G_1_ phase was 39.76% (Lane 6 in Figure 6). This supplementary experiment suggested that SP600125 might induce blocks of cell cycle by down-regulating the expression of MAD2.

### Preparing Fish Chromosomes for Karyotype Analysis by SP600125 Pretreatment *in vitro* and *in vivo*

**Figure 7:**
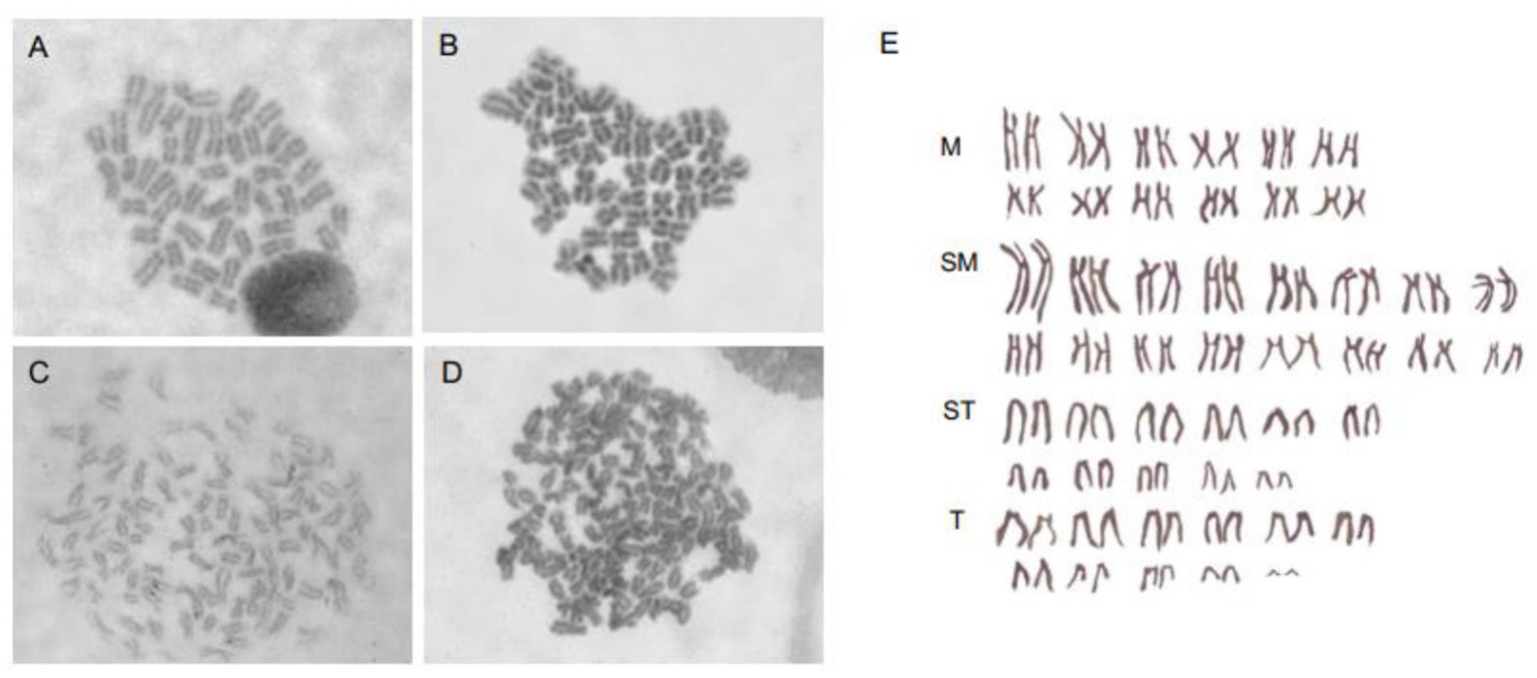
Chromosomes preparation by SP600125 pretreatment *in vitro* and *in vivo*. **(A,B)** Chromosome splitting phases of blunt snout bream from caudal fin cell and kidney tissue, respectively. **(C,D)** Chromosome splitting phases of red crucian carp from caudal fin cell and kidney tissue, respectively. **(E)** Chromosomal karyotype of red crucian carp.

Taking into account that SP600125 could block the cell cycle progression at prometaphase of mitosis, we now present a new method of preparing fish chromosomes for karyotype analysis. Chromosome preparation was made from two kinds of fish samples, the cultured cells *in vitro* and the kidney tissue *in vivo*. The cultured fish cells were first treated by 100 μmol/L SP600125 for 22–28 h and digested by trypsin, and then they were collected by centrifugation for preparing chromosome. For the fish kidney tissue, PHA was injected into the fish three times, and then the fish was injected with SP600125. As shown in Figure 7, not only for the samples from the blunt snout bream (2n = 48) but also for those from the red crucian carp (2n = 100), the split chromosomes had clear morphology and moderate length.

## Discussion

Endoreduplication is a variant of cell proliferation with replication of nuclear genome, where mitosis fails. There are two types of endoreduplication: endocycling and endomitosis. With respect to endocycling, the cell proliferation skips the mitotic phase (Ma et al., 1996; Rieder and Maiato, 2004; Lilly and Duronio, 2005), whereas for endomitosis, the cells can enter into mitosis, but the distribution of chromosomes fails (Vitrat et al., 1998). In this study, flow cytometry analysis has indicated that for the SP600125-treated fish cells, the process of mitosis was also blocked, and the tetraploid appeared (Figure 1). Cytological observation further indicated that for the SP600125-treated fish cells, the cell cycle progression was arrested at prometaphase of mitosis (Figure 2). In other words, endomitosis seemed to display for these cells.

Note that the presented result in this article is inconsistent with that reported by Kim et al. (2010). Actually, they found that SP600125 could prevent the entry of cells (HCT116, human colorectal carcinoma cells) into mitosis and induce endoreplication of DNA from G_2_ phase. Thus, it was suggested the endocycling was caused (Kim et al., 2010). Significant difference between Kim's experiments and ours might be the reason of causing the above distinct results. For example, in Kim et al. (2010), 20 μM SP600125 was used to treat the HCT116 cells. In contrast, we used 100 μM SP600125 to treat the fish cells (Zhou et al., 2016; Mo et al., 2019). It has been reported that the concentration of SP600125 is a crucial factor to the experimental results (Bennett et al., 2001; Chang and Karin, 2001), where SP600125 was regarded to be a reversible ATP-competitive inhibitor with >20-fold selectivity over a range of kinases. It has been found that JNK1/2 and JNK3 with IC50 of 40 and 90 nM were inhibited, where the selective inhibition of JNK was more than 300 times higher than that of ERK1 and p38 MAPK (Bennett et al., 2001; Han et al., 2001; Vaishnav et al., 2003; Yarza et al., 2016; Gkouveris and Nikitakis, 2017). In conclusion, it needs further investigation to exactly answer whether SP600125 induces endocycling and endomitosis in a dose-dependent manner or not.

The experimental results have demonstrated that the expression levels of p53 signaling pathway genes were significantly up-regulated in the SP600125-treated cells (i.e., *mdm2, p53, p27, p21, socs3, gadd45*α, *14-3-3*σ), but those of the cyclin-Cdk complex genes (i.e., *cyclin B1/3* and *cdc2*) and the SAC genes (i.e., *mad2, cdc20*) were down-regulated (Figures 3, 5). Furthermore, the proportion of the 4n peak cells in the p53 inhibitor PFT-α group was much smaller than that of the cells in the SP600125 group, suggesting that the PFT-α might affect mitosis arrest in the SP600125-treated cells (Figure 4). In addition, overexpression of MAD2 could alleviate SAC arrest in the SP600125-treated cells (Figure 5). Summarily, it follows from our results that the enhanced p53 pathway and the weakened SAC associated with the SP600125 treatment might play a critical role in blocking the cell cycle of fish cells. It has been reported that DNA damage could cause p53 expression and activation of downstream target genes, such as *p21, Gadd45*α, and *14-3-3*σ (Fujiwara et al., 2005; Purvis et al., 2012). The *p21* and *14-3-3*σ could inhibit cyclin-dependent protein kinase activity, whereas Gadd45α promoted DNA repair (Waldman et al., 1995; Hermeking et al., 1997; Wang et al., 1999). The p53 could down-regulate the expression levels of *cdc2* and *cyclinB1*, induce growth arrest, and activate the process of cell polyploidization (Aylon and Oren, 2011). It was also suggested that as a cellular surveillance, the SAC could ensure faithful chromosome segregation in mitosis (Musacchio and Salmon, 2007; Musacchio, 2015). In fact, MAD2 can mediate the spindle checkpoint by blocking the function of CDC20 to recruit substrates to the anaphase-promoting complex (APC) (Taylor et al., 2004; Musacchio, 2015). Down-regulated expression of MAD2 could result in weakening SAC, which leads to chromosomal instability and polyploidy in cancer cells (Luo et al., 2000; Michel et al., 2001, 2004; Wang et al., 2010).

Our immunofluorescence staining observation has showed that the spindle microtubules were clearly visible in the SP600125-treated fish cells (Figure 2). It seems that SP600125 could not inhibit the spindle microtubule assembly, but colchicine can do (Herdman et al., 2016). Since SP600125 can arrest cell cycle at prometaphase of mitosis, we have selected SP600125 to pretreat the fish cells in chromosomes preparation for karyotype analysis. It was also shown that the chromosome specimens prepared by SP600125 were of clear morphology and moderate length *in vitro* and *in vivo*. Therefore, the presented method in this article forms a new way of chromosomes preparation for karyotype analysis by replacing colchicine with SP600125.

As a drug with lower toxicity, SP600125 has been used to treat cancer, induce pluripotent stem cells, and breed polyploidy (Kim et al., 2014; Wei et al., 2014; Yao et al., 2014; Ou et al., 2016; Zhou et al., 2016). About 40% of all human tumors undergo a tetraploid intermediate state (Zack et al., 2013). The transition from diploid cells to tetraploid ones is a critical event in the early stages of tumorigenesis (Ganem et al., 2007; Gordon et al., 2012; Jemaà et al., 2013; Ohshima and Seyama, 2013, 2017; Kuznetsova et al., 2015). Therefore, the proposed way of tetraploidization using the SP600125 treatment might be helpful to further explore the molecular mechanism of polyploidy in tumorigenesis.

## Conclusions

In this study, we have proposed a new method of chromosomes preparation for karyotype analysis by SP600125 *via* enhancement of endomitosis in fish. Specifically, by flow cytometry analysis and cytological observation, we have found that in the SP600125-treated fish cells, the cell cycle progression can be arrested at prometaphase of mitosis and cell polyploidization displays, which might be caused by enhancement of the p53 signaling pathway and weakening of the SAC. Therefore, the obtained results might provide important implications to further explore the molecular mechanism of polyploidy in tumorigenesis and cancer treatment.

## Data Availability Statement

Publicly available datasets were analyzed in this study. This data can be found here: The mRNA sequencing (seq) data of caudal fin cells of crucian carp and SP600125-treated cells from the NCBI SRA database (SRR7640866, SRR7640867, SRR9964682, and SRR9964683).

## Ethics Statement

The animal study was reviewed and approved by Institutional Animal Care and Use Committee of Hunan Normal University.

## Author Contributions

YX, WX, and YM designed the experiments and organized and wrote the manuscript. YM, WX, YH, WF, YF, SC, and YX carried out the experiments. YM, WX, GH, JL, WL, LP, and YX conducted the statistical analysis and discussion.

## Conflict of Interest

The authors declare that the research was conducted in the absence of any commercial or financial relationships that could be construed as a potential conflict of interest.

## References

[B1] Aylon Y Oren M. (2011). p53: guardian of ploidy. Mol. Oncol. 5, 315–323. 10.1016/j.molonc.2011.07.007 21852209 PMC3164924

[B2] Bennett B. L. Sasaki D. T. Murray B. W. Leary E. C. O'Sakata S. T. (2001). SP600125, an anthrapyrazolone inhibitor of Jun N-terminal kinase. Proc. Natl. Acad. Sci. U.S.A. 98, 13681–13686. 10.1073/pnas.251194298 11717429 PMC61101

[B3] Chang L. Karin M. (2001). Mammalian MAP kinase signalling cascades. Nature 410, 37–40. 10.1038/35065000 11242034

[B4] Chen M. Qian C. Bi L. L. Zhao F. Zhang G. Y. Wang Z. Q.. (2015). Enrichment of cardiac differentiation by a large starting number of embryonic stem cells in embryoid bodies is mediated by the Wnt11-JNK pathway. Biotechnol. Lett. 37, 475–481. 10.1007/s10529-014-1700-5 25312921

[B5] Dhanasekaran D. N. Reddy E. P. (2017). JNK-signaling: a multiplexing hub in programmed cell death. Genes Cancer 8, 682–694. 10.18632/genesandcancer.155 29234486 PMC5724802

[B6] Fujiwara T. Bandi M. Nitta M. Ivanova E. V. Bronson R. T. Pellman D. (2005). Cytokinesis failure generating tetraploids promotes tumorigenesis in p53-null cells. Nature 437, 1043–1047. 10.1038/nature04217 16222300

[B7] Ganem N. J. Storchova Z. Pellman D. (2007). Tetraploidy, aneuploidy and cancer. Curr. Opin Genet. Dev. 17, 157–162. 10.1016/j.gde.2007.02.011 17324569

[B8] Gkouveris I. Nikitakis N. G. (2017). Role of JNK signaling in oral cancer: a mini review. Tumor Biol. 39:1010428317711659. 10.1177/1010428317711659 28639904

[B9] Gordon D. J. Resio B. Pellman D. (2012). Causes and consequences of aneuploidy in cancer. Nat. Rev. Genet. 13, 189–203. 10.1038/nrg3123 22269907

[B10] Hai Y. Y. Chun L. W. Xiang Y. Qianh H. B. Chun H. Q. Meng B. L.. (2019). SP600125 enhances C-2-induced cell death by the switch from autophagy to apoptosis in bladder cancer cells. J. Exp. Clin. Cancer Res. 38:448. 10.1186/s13046-019-1467-6 31685029 PMC6829950

[B11] Han H. Boyle D. Chang L. Bennett B. Karin M. Manning A.. (2001). c-Jun N-terminal kinase is required for metalloproteinase expression and joint destruction in inflammatory arthritis. J. Clin. Invest. 108, 73–81. 10.1172/JCI12466 11435459 PMC209341

[B12] Herdman C. A. Strecker T. E. Tanpure R. P. Chen Z. Winters A. Gerberich J.. (2016). Synthesis and biological evaluation of benzocyclooctene-based and indene-based anticancer agents that function as inhibitors of tubulin polymerization. Medchemcomm. 7, 2418–2427. 10.1039/C6MD00459H 28217276 PMC5308454

[B13] Hermeking H. Lengauer C. Polyak K. He T. C. Zhang L. Thiagalingam S.. (1997). 14-3-3sigma is a p53-regulated inhibitor of G2/M progression. Mol Cell. 1, 3–11. 10.1016/S1097-2765(00)80002-7 9659898

[B14] Jemaà M. Galluzzi L. Kepp O. Castedo M. Rello-Varona S. Vitale I.. (2013). A method for the graphical presentation of complex cell cycle alterations. Cell Cycle 12, 183–190. 10.4161/cc.23046 23255111 PMC3570510

[B15] Kim J. A. Lee J. Margolis R. L. Fotedar R. (2010). SP600125 suppresses Cdk1 and induces endoreplication directly from G2 phase, independent of JNK inhibition. Oncogene 29, 1702–1716. 10.1038/onc.2009.464 20062077 PMC3145494

[B16] Kim J. H. Chae M. Choi A. R. Sik kim H. Yoon S. (2014). SP600125 overcomes antimitotic drug-resistance in cancer cells by increasing apoptosis with independence of P-gp inhibition. Eur. J. Pharmacol. 15, 141–147. 10.1016/j.ejphar.2013.11.026 24333214

[B17] Kitagawa R. Rose A. M. (1999). Components of the spindle-assembly checkpoint are essential in *Caenorhabditis elegans*. Nat. Cell Biol. 1, 514–521. 10.1038/70309 10587648

[B18] Komarov P. G. Komarova E. A. Kondratov R. V. Tselkov K. C. Coon J. S. Chernov M. V.. (1999). A chemical inhibitor of p53 that protects mice from the side effects of cancer therapy. Science 285, 1733–1737. 10.1126/science.285.5434.1733 10481009

[B19] Kook S. H. Jeon Y. M. Lim S. S. Jang M. J. Cho E. S. Lee S. Y.. (2013). Fibroblast growth factor-4 enhances proliferation of mouse embryonic stem cells via activation of c-Jun signaling. PLoS ONE 8:e71641. 10.1371/journal.pone.0071641 23967228 PMC3742512

[B20] Kuznetsova A. Y. Seget K. Moeller G. K. de Pagter M. S. de Roos J. A. Dürrbaum. (2015). Chromosomal instability, tolerance of mitotic errors and multidrug resistance are promoted by tetraploidization in human cells. Cell Cycle 14, 2810–2820. 10.1080/15384101.2015.1068482 26151317 PMC4614355

[B21] Li J. Y. Huang J. Y. Xing B. Ren K. W. Li M. Wei D.. (2012). SP600125, a JNK inhibitor, suppresses growth of JNK-inactive glioblastoma cells through cell-cycle G_2_/M phase arrest. Die Pharmazie 67, 942–946. 10.1691/ph.2012.1849 23210245

[B22] Lilly M. A. Duronio R. J. (2005). New insights into cell cycle control from the Drosophila endocycle. Oncogene 24, 2765–2775. 10.1038/sj.onc.1208610 15838513

[B23] Luo X. Fang G. Coldiron M. Lin Y. Yu H. Kirschneet M. W.. (2000). Structure of the Mad2 spindle assembly checkpoint protein and its interaction with Cdc20. Nat. Struct. Biol. 7, 224–229. 10.1038/73338 10700282

[B24] Ma D. C. Sun Y. H. Chang K. Z. Zuo W. (1996). Developmental change of megakaryocyte maturation and DNA ploidy in human fetus. Eur. J. Haematol. 57, 121–127. 10.1111/j.1600-0609.1996.tb01349.x 8856088

[B25] Michel L. Diaz-Rodriguez E. Narayan G. Hernando E. Murty V. V. V. S. Benezraet R. (2004). Complete loss of the tumor suppressor MAD2 causes premature cyclin B degradation and mitotic failure in human somatic cells. Proc. Natl. Acad. Sci. U.S.A. 101, 4459–4464. 10.1073/pnas.0306069101 15070740 PMC384769

[B26] Michel L. S. Liberal V Chatterjee A. Kirchwegger R. Pasche B. Gerald W.. (2001). MAD2 haplo-insufficiency causes premature anaphase and chromosome instability in mammalian cells. Nature 409, 355–359. 10.1038/35053094 11201745

[B27] Mili D. Abid K. Rjiba I. Kenani A. (2016). Effect of SP600125 on the mitotic spindle in HeLa Cells, leading to mitotic arrest, endoreduplication and apoptosis. Mol Cytogenet. 9:86. 10.1186/s13039-016-0296-y 27924151 PMC5123282

[B28] Mo Y. Fan Y Fu W. Xu W. Chen S Wen Y.. (2019). Acute immune stress improves cell resistance to chemical poison damage in SP600125-induced polyploidy of fish cells *in vitro*. Fish Shellfish Immunol. 84, 656–663. 10.1016/j.fsi.2018.10.063 30393156

[B29] Moon D. O. Choi Y. H. Kim G. Y. (2011). Role of p21 in SP600125-induced cell cycle arrest, endoreduplication, and apoptosis. Cell Mol Life Sci. 68, 3249–3260. 10.1007/s00018-011-0626-5 21311948 PMC11114892

[B30] Moon D. O. Kim M. O. Kang C. H. Lee J. D. Choi Y. H. Kim G. Y. (2009). JNK inhibitor SP600125 promotes the formation of polymerized tubulin, leading to G_2_/M phase arrest, endoreduplication, and delayed apoptosis. Exp. Mol. Med. 41, 665–677. 10.3858/emm.2009.41.9.073 19478553 PMC2753660

[B31] Musacchio A. (2015). The molecular biology of spindle assembly checkpoint signaling dynamics. Curr. Biol. 25, R1002–R1018. 10.1016/j.cub.2015.10.050 26485365

[B32] Musacchio A. Salmon E. D. (2007). The spindle-assembly checkpoint in space and time. Nat. Rev. Mol. Cell Biol. 8, 379–393. 10.1038/nrm2163 17426725

[B33] Nakaya K. Ooishi R. Funaba M. Murakami M. (2009). A JNK inhibitor SP600125 induces defective cytokinesis and enlargement in P19 embryonal carcinoma cells. Cell Biochem. Funct. 27:468–472. 10.1002/cbf.1597 19711443

[B34] Nicklas R. B. (1997). How cells get the right chromosomes. Science 275, 632–637. 10.1126/science.275.5300.632 9005842

[B35] Ohshima S. Seyama A. (2013). Establishment of proliferative tetraploid cells from normal human fibroblasts. Front Oncol. 3:198. 10.3389/fonc.2013.00198 23914348 PMC3730083

[B36] Ohshima S. Seyama A. (2017). Establishment of proliferative tetraploid cells from nontransformed human fibroblasts. J. Vis. Exp. 119:55028 10.3791/55028 PMC5408595 28117785

[B37] Ou D. Wang Q. Huang Y. Zeng D. Wei T. Ding L.. (2016). Co-culture with neonatal cardiomyocytes enhances the proliferation of iPSC-derived cardiomyocytes via FAK/JNK signaling. BMC Dev. Biol. 16:11. 10.1186/s12861-016-0112-2 27141946 PMC4855360

[B38] Peng L. Zhou Y. Xu W. Jiang M. Li H. Long M.. (2019). Generation of stable induced pluripotent stem-like cells from adult zebra fish fibroblasts. Int. J. Biol. Sci.15, 2340–2349. 10.7150/ijbs.34010 31595152 PMC6775306

[B39] Purvis J. E. Karhohs K. W. Mock C. Atchelor E. Loewer A. Lahav G. (2012). p53 dynamics control cell fate. Science 336:1440–1444. 10.1126/science.1218351 22700930 PMC4162876

[B40] Ren L. Lu J. Fan Y. Hu Y. Li J. Xiao Y.. (2020). Expression profile analysis of the cell cycle in diploid and tetraploid carassius auratus red var. Front. Genet. 11:203. 10.3389/fgene.2020.00203 32256518 PMC7089929

[B41] Rieder C. L. Maiato H. (2004). Stuck in division or passing through: what happens when cells cannot satisfy the spindle assembly checkpoint. Dev Cell. 7, 637–651. 10.1016/j.devcel.2004.09.002 15525526

[B42] Taylor S. S. Scott M. I. F. Holland A. J. (2004). The spindle checkpoint: a quality control mechanism which ensures accurate chromosome segregation. Chromosome Res. 12, 599–616. 10.1023/B:CHRO.0000036610.78380.51 15289666

[B43] Vaishnav D. Jambal P. Reusch J. E. Pugazhenthi S. (2003). SP600125, an inhibitor of c-jun N-terminal kinase, activates CREB by a p38 MAPK-mediated pathway. Biochem. Biophys. Res. Commun. 307, 855–860. 10.1016/S0006-291X(03)01287-7 12878189

[B44] Vitrat N. Cohen-Solal K. Pique C. Le C. J. P. Norol F. Larsen A. K.. (1998). Endomitosis of human megakaryocytes are due to abortive mitosis. Blood 91, 3711–3723. 10.1006/bcmd.1998.0187 9573008

[B45] Waldman T. Kinzler K. W. Vogelstein B. (1995). p21 is necessary for the p53-mediated G1 arrest in human cancer cells. Cancer Res. 55, 5187–5190. 7585571

[B46] Wang L. Yin F. Du Y. Chen B. Liang S. H. Zhang Y. G.. (2010). Depression of MAD2 inhibits apoptosis and increases proliferation and multidrug resistance in gastric cancer cells by regulating the activation of phosphorylated survivin. Tumour Biol. 31, 225–232. 10.1007/s13277-010-0036-6 20440596

[B47] Wang X. W. Zhan Q. Coursen J. D. Khan M. A. Kontny H. U. Yu L.. (1999). GADD45 induction of a G_2_/M cell cycle checkpoint. Proc. Natl. Acad. Sci. U.S.A. 96, 3706–3711. 10.1073/pnas.96.7.3706 10097101 PMC22358

[B48] Wei Z. Z. Yu S. P. Lee J. H. Chen D. Taylor T. M. Deveau T. C.. (2014). Regulatory role of the JNK-STAT1/3 signaling in neuronal differentiation of cultured mouse embryonic stem cells. *Cell Mol*. Neurobiol. 34, 881–893. 10.1007/s10571-014-0067-4 24913968 PMC11488891

[B49] Yao K. Ki M. O. Chen H. Cho Y. Y. Kim S. H. Yu D.. (2014). JNK1 and 2 play a negative role in reprogramming to pluripotent stem cells by suppressing Klf4 activity. Stem Cell Res. 12, 139–152. 10.1016/j.scr.2013.10.005 24211391

[B50] Yarza R. Vela S. Solas M. Ramirez M. J. (2016). C-Jun N-terminal kinase (JNK) signaling as a therapeutic target for Alzheimer's disease. Front. Pharmacol. 6:321. 10.3389/fphar.2015.00321 26793112 PMC4709475

[B51] Zack T. I. Schumacher S. E. Carter S. L. Cherniack A. D. Saksena G. Tabak B.. (2013). Pan-cancer patterns of somatic copy number alteration. Nat. Genet. 45, 1134–1140. 10.1038/ng.2760 24071852 PMC3966983

[B52] Zhou Y. Wang M. Jiang M. Peng L. Wan C. Liu J.. (2016). Autotetraploid cell Line induced by SP600125 from crucian carp and its developmental potentiality. Sci. Rep. 6:21814. 10.1038/srep21814 26898354 PMC4761888

