# Supplementary material for: A New Method for Chromosomes Preparation by ATP-Competitive Inhibitor SP600125 via Enhancement of Endomitosis in Fish
Source: Front Bioeng Biotechnol. 2021 Jan 13;8:606496. doi: 10.3389/fbioe.2020.606496 (PMC7838586; doi:10.3389/fbioe.2020.606496)
Supplement: Supplementary file 1 [file Presentation_1.pdf]

Table S1 Primer sequences used for qRT-PCR.

genes	primers	Sequences (5' to 3')	genes	primers	Sequences (5' to 3')
mdm2	mdm2+	ACACGACTGACTGAATGGG	ccnb1	ccnb1+	CCAGCAGCGAGAATCAGA
	mdm2-	TGTCAAAGTCGCCCCAAGAA		ccnb1-	AGCCACAACCTCCACCTT
p53	p53+	GTGTGGCGGTTCCCTATGAA	ccnb3	ccnb3+	TGCGGTGTGTCTGTTTCAGT
	p53-	TAGTAGAGGGGGTCTTGCCC		ccnb3-	GATACGCTGACCAACCCCAA
p27	p27+	CAAATAATCGTGGTTCCC	bub1	bub1+	CTGTACTTCACCGTCTGC
	p27-	CTACGAGTGGCAGGAGGT		bub1-	CACTGGAAGCCTGAAGTC
gadd45α	gadd45α+	GTCCACTTTTATCTTCCCA	mad2	mad2+	TGGAGACTAATGAGGTGCTG
	gadd45α-	GCCTACTATTACTCTGCCG		mad2-	CAAGAAGGTGACTGTGGC
socs3	socs3+	GACCACAGGCACCAAAAACC	cdc20	cdc20+	GCTGGTAGGCTTGGGTTTCT
	socs3-	CGCAGTAGCTCCAAAGGGAT		cdc20-	TGTCTGGTCACGGTTTTGCT
cdc2	cdc2+	TCTTCCAGCCAGTAACC	securin	securin+	CGAGTTTACCAGGAAGGA
	cdc2-	GCCACCCAATAAACAAAT		securin-	CCAGAGCGGTTGAATGTG
β-actin	β-actin+	CGAGAAGATGACCCAGATCA			
	β-actin-	GATCTTCATGAGGTAGTCAG			

Table S2 Analysis of significant difference expressed genes related to cell cycle-regulation (SP600125-treated 48h VS control cells)

Seq- ID	Gene name	SP600125-treated Vs control log2 fold Change	Adjusted P-value	Note
c81783_g4	p53	1.52	6.46E-07	Cellular tumor antigen p53
c88250_g2	mdm2	1.94	5.48E-10	E3 ubiquitin-protein ligase Mdm2
c80659_g3	p21(CDKN1)	1.2	2.07E-03	cyclin-Dependent Kinase Inhibitor 1A
c87763_g3	p27(CDKN1B)	1.13	1.96E-03	cyclin-Dependent Kinase Inhibitor27
c59135_g1	p15(CDKN2B)	1.4	1.93E-04	Cyclin-dependent kinase inhibitor 2A
c80044_g1	gadd45a	2.28	4.59E-08	growth arrest and DNA damage-inducible protein
c50597_g1	14-3-3σ	-1.39	7.63E-04	14-3-3sigma protein
c86455_g1	cdc2	-2	6.47E-04	cyclin-dependent kinase 2
c89577_g2	cyclinB1	-1.92	1.15E-03	cyclin-dependent kinase B1
c88235_g1	cyclinB3	-2.29	4.23E-04	cyclin-dependent kinase B3
c87470_g1	cyclinA	-2.16	6.72E-04	Cyclin-A2
c86200_g1	p48	2.43	8.16E-13	Interferon consensus sequence-binding protein
c88655_g1	rbl2	1.26	2.26E-05	retinoblastoma like protein 2
c86566_g1	bub1	-1.76	7.75E-06	mitotic checkpoint serine/threonine-protein kinase
c81863_g1	mad2	-1.63	1.51E-03	spindle assembly checkpoint protein
c86627_g1	cdc20	-1.82	1.87E-03	cell division cycle protein 20 homolog
c87478_g1	securin	2.05	2.16E-05	Securin
c86455_g1	cdk1	-2	6.47E-04	cyclin-dependent kinase 1
c87293_g1	pkl1	-1.95	4.90E-04	Polo-Like 1
c90774_g1	cdc14	1.26	1.81E-04	cell divisioncycle gene14A
c80357_g1	cks1	-1.69	1.57E-03	CDC28 protein kinase 2
c86804_g1	socs3	1.29	1.80E-04	suppressor of cytokine signaling 3b
c91500_g1	smc4	-1.81	1.02E-03	Structural maintenance of chromosomes protein 4
c88581_g3	rassf5	1.37	4.27E-05	ras association domain-containing protein 5-like isoform X1
c83067_g1	pttg	-1.91	1.88E-03	pituitary tumor-transforming 1
c93217_g2	esp1	-2.25	3.30E-04	Extra spindle poles-like 1 protein
c89653_g1	yeg1	-1.64	4.33E-03	non-SMC condensin I complex, subunit G
c86622_g1	bm1	-1.74	4.33E-03	non-SMC condensin I complex, subunit H
c91486_g2	frizzled	2.33	5.19E-12	Frizzled homolog 7a

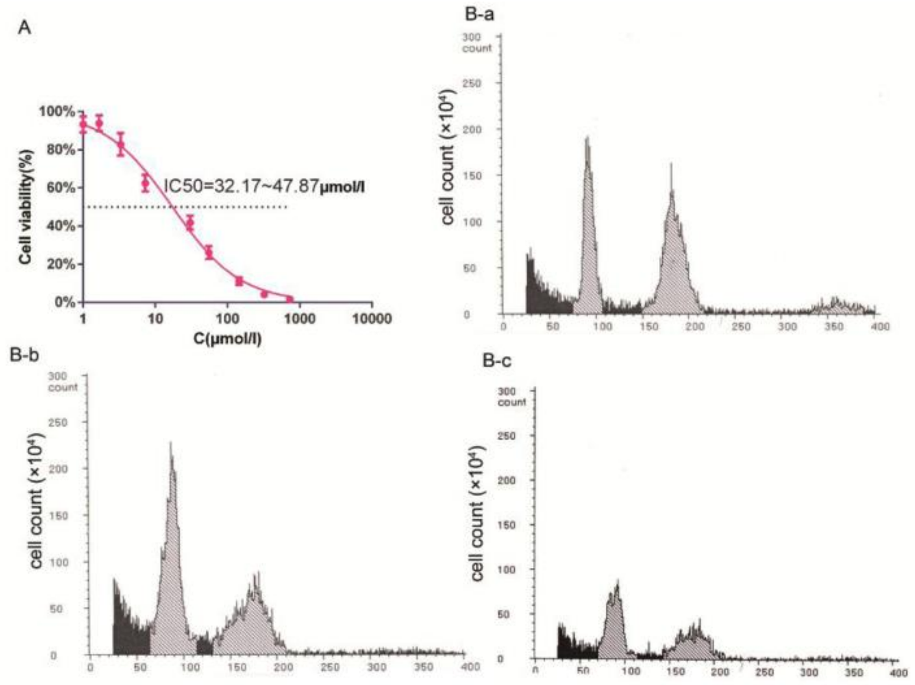

Figure S1 Effects of PFT- α on SP600125-induced polyploidization in diploid crucial carp cells. (A) The cytotoxicity of PFT-α was detected by cell counting kit-8 (CCK-8). (B) Diploid crucial carp cells treated with the indicated concentrations of PFT-α were determined by flow cytometry examination. The “a”, “b”, and “c” represent 30, 40, and 50 μM concentrations of PFT-α, respectively.

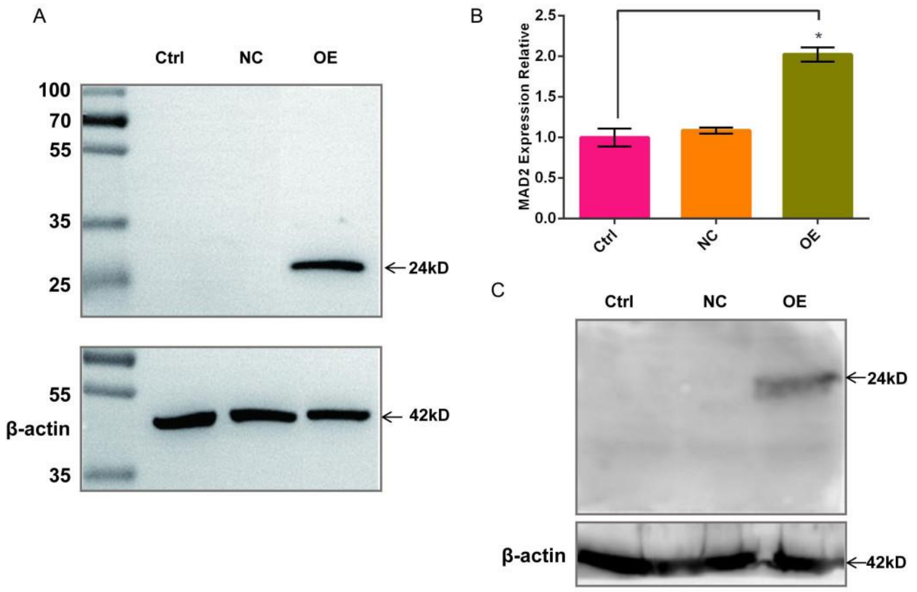

Figure S2 Overexpression of MAD2 by recombinant adenovirus pAd-MAD2-GFP-3Flag fusion protein in fin cells. (A) Expression lever of the recombinant adenovirus pAd-MAD2--GFP-3Flag fusion protein in 293T cells as detected by Western blot. The anti-Flag primary antibody was used at a 1:4000 dilution, while the Marker was diluted 1: 3000. (B) The mRNA expression of *mad2* gene in fin cells transfected with Ad-MAD2 recombinant protein. (C) The protein expression of *mad2* gene in fin cells transfected with Ad-MAD2 recombinant protein. The “Ctrl”, “NC”, and “OE” represent the blank control group, negative control group (transfected pAd-mCMV-GFP-3-Flag-pA), and MAD2-OE group (transfected pAd-mCMV-MAD2-GFP-3-Flag-pA), respectively. Values were normalized by β-actin expression level. Each bar represents the mean ± SD of three independent experiments (*P < 0.05).
